# Exploring the antimicrobial, anti-inflammatory, antioxidant, and immunomodulatory properties of *Chrysanthemum morifolium* and *Chrysanthemum indicum*: a narrow review

**DOI:** 10.3389/fphar.2025.1538311

**Published:** 2025-03-19

**Authors:** Yuqing Liang, Tengwen Liu, Dong Wang, Qingquan Liu

**Affiliations:** ^1^ School of Basic Medical Sciences, Chengdu University of Traditional Chinese Medicine, Chengdu, China; ^2^ Beijing Hospital of Traditional Chinese Medicine, Capital Medical University, Beijing, China; ^3^ Beijing Institute of Chinese Medicine, Beijing, China; ^4^ Beijing Key Laboratory of Basic Research with Traditional Chinese Medicine on Infectious Diseases, Beijing, China

**Keywords:** *Chrysanthemum morifolium*, *Chrysanthemum indicum*, anti-infection, major compounds, pharmacological effects

## Abstract

Infectious diseases continue to be a major global public health concern, which is exacerbated by the increasing prevalence of antimicrobial resistance. This review investigates the potential of herbal medicine, particularly *Chrysanthemum morifolium* (CM) and *Chrysanthemum indicum* (CI), in addressing these challenges. Both herbs, documented in traditional Chinese medicine (TCM) and the Pharmacopoeia of the People’s Republic of China (2020 edition), are renowned for their heat-clearing and detoxifying properties. Phytochemical studies reveal that these botanicals contain diverse bioactive compounds, including flavonoids, terpenoids, and phenylpropanoids, which exhibit antimicrobial, anti-inflammatory, and antioxidant properties, among other effects. Comparative analysis reveals that distinct compound profiles and differential concentrations of core phytochemicals between CM and CI may lead to differentiated therapeutic advantages in anti-infective applications. By systematically examining their ethnopharmacological origins, phytochemical fingerprints, and pharmacological mechanisms, this review highlights their synergistic potential with conventional antimicrobial therapies through multi-target mechanisms, proposing novel integrative approaches for global health challenges.

## 1 Introduction

Infectious diseases continue to pose a persistent and significant threat to global public health, contributing substantially to morbidity and mortality worldwide. The rise of antimicrobial resistance has further complicated the management of these diseases, highlighting the urgent need for alternative therapeutic strategies (Sangeetha Vijayan et al., 2024). Botanical drug, with its extensive historical and cultural heritage, presents a promising avenue for addressing both infectious diseases and the challenges posed by antimicrobial resistance.

*Chrysanthemum morifolium* (CM, known as “Juhua” in China) refers to the flower head of *C. morifolium* Ramat. Originating from China, CM has been utilized for over 3,000 years, predominantly as a dietary flower tea for health maintenance and as an integral component of traditional Chinese medicine (TCM) (Yuan et al., 2020). CM is listed in the Pharmacopoeia of the people’s Republic of China (ChP) as a significant botanical drug with properties that include dispersing wind-heat, clearing liver fire, brightening the eyes, and detoxifying (Commission CP, 2020). *Chrysanthemum indicum* (CI, known as “Yeju” in China) is the flower head of *C. indicum* Linné. According to the 2020 edition of ChP, CI is recognized for its properties in clearing heat, detoxifying, purging fire, and pacifying the live (Commission CP, 2020). The similar names and appearances of the two botanical drugs frequently cause confusion and misidentification. Although both CM and CI belong to the Compositae family, their plant morphology and phytochemical compositions differ significantly. In TCM, CI is considered more effective for heat-clearing and detoxifying, while CM exhibits a broader range of therapeutic functions. Nonetheless, both botanical drugs show potential in the treatment of infectious diseases.

The pharmacological potential of CM and CI in infectious disease management is underscored by their rich phytochemical composition. These plants contain a plethora of compounds, including flavonoids (Miyazawa and Hisama, 2003; Wang et al., 2010; Wu et al., 2015; Yang et al., 2019a), terpenoids (Commission CP, 2020; Liu et al., 2017), phenolic acids (Yang et al., 2019b; Gao et al., 2008), and others (Guo et al., 2010). Notably, these compounds demonstrate antibacterial (Park and Kang, 2021; Zhang et al., 2024; Wang et al., 2020), antiviral (Shen et al., 2018; Boudou et al., 2024), anti-inflammatory (Boudou et al., 2024; Hamad et al., 2023), antioxidant effects (Boudou et al., 2024; Han et al., 2018), among others (Chen et al., 2024; Yang et al., 2017; Xie et al., 2009; Sun et al., 2012). Due to the differences in the types of compounds and the concentrations of the main compounds between the two, they may have different characteristics and advantages in anti-infection treatment. However, there has been no comprehensive summary or comparison of the effects of CM and CI in the treatment of infectious diseases.

This article aims to explore the main compounds and potential mechanisms by which CM and CI play a role in the treatment of infectious diseases. By delving into the ethnopharmacological origins, major phytochemical compounds, and associated pharmacological activities of these botanicals, we aim to elucidate their potential in complementing conventional anti-infection therapies and addressing the challenges of antimicrobial resistance. Finally, the paper summarizes the current research status and discusses future prospects of CM and CI in the context of infectious diseases.

## 2 Ethnopharmacology

### 2.1 Botany, description and distribution

Both CM and CI belong to the Compositae family and are utilized both medicinally and for environmental applications. The dried flower heads of CM are commonly utilized as medicinal parts for treatment, while both the dried flower heads and whole plants of CI serve as primary medicinal components for treating various diseases. To better differentiate between CM and CI, we conducted a macroscopic identification, as detailed in Table 1.

**TABLE 1:** The macroscopic identification of CM and CI.

Macroscopic	CM	CI
Appearance	Conical, inverted conical, flat spherical, irregular spheroidal, disc-shaped	Spheroid
Size	2.5–20 cm in diameter	0.25–1.3 cm in diameter
Surface feature	Involucrum composed of 3–4 layers, ovate to oval in shape, herbaceous in texture, colored yellow-green to brown-green. The outer surface is pilose, while the margin is membranous. The receptacle is hemispherical. Ligulate flowers are arranged in several layers, female, located peripherally, off-white in color, stiff and straight with longitudinal folds, and scattered with golden glands. Numerous tubular flowers, which are bisexual and centrally located, are hidden by the ligulate flowers, appearing yellow with apically 5-dentate corollas. Achenes are undeveloped and glabrous	Involucrum composed of 4–5 bracts. The outer bracts are ovate or striate, colored grayish-green to pale brown, often bearing white hairs, with membranous margins. The inner bracts are elongated and oval-shaped, membranous, and have a glabrous outer surface. Residual pedicels are present at the base of the involucre. Ligulate flowers range from yellow to brownish, exhibiting wrinkled and curled morphology. Numerous tubular flowers are present, characterized by a dark yellow color
Texture	Lightweight, with a soft texture and crispness when dry	Lightweight
Smell	Fresh	Fragrant
Taste	Sweet with a subtle bitterness	Bitter

CM (Juhua) has been utilized in China for over 3,000 years, with historical records dating back to the Qin and Han dynasties (Liang et al., 2014). It was introduced to Japan during the Tang Dynasty (AD 710–784) as a highly regarded spice and subsequently spread Europe and the United States in the 17th century (Li, 1993). The genus CM comprises 41 species, which are widely distributed across Asia, including Mongolia, Russia, China, Japan, and Korea, as well as eastern Europe (Chen et al., 2020; Committee FoCE, 1983). In China, excluding Tibet and the Northwest regions, approximately 21 species are predominantly found in humid areas at middle and low altitudes (Chen et al., 2020). The botanical morphological characteristics of CM are described as follows: 1) Life form: Perennial herb. 2) Stem: Erect, either branched or unbranched, covered with pubescent. 3) Leaves: Ovate to lanceolate in shape, measuring 5–15 cm in length, pinnately lobed or semi-lobed, short-petioled, and white pubescent on the underside. 4) Flowers: The capitulum ranges from 2.5 to 20 cm in diameter. The involucrum consists of multiple layers, with the outer layer being pilose. The ray florets exhibit a variety of colors, whereas the disk florets are yellow. 5) Fruits: Achenes measure approximately 1.3 mm in length, slightly pointed at the apex, flat-wedge shaped, longitudinally ribbed on the surface, and brown in color (Committee FoCE, 1983).

**FIGURE 1:**
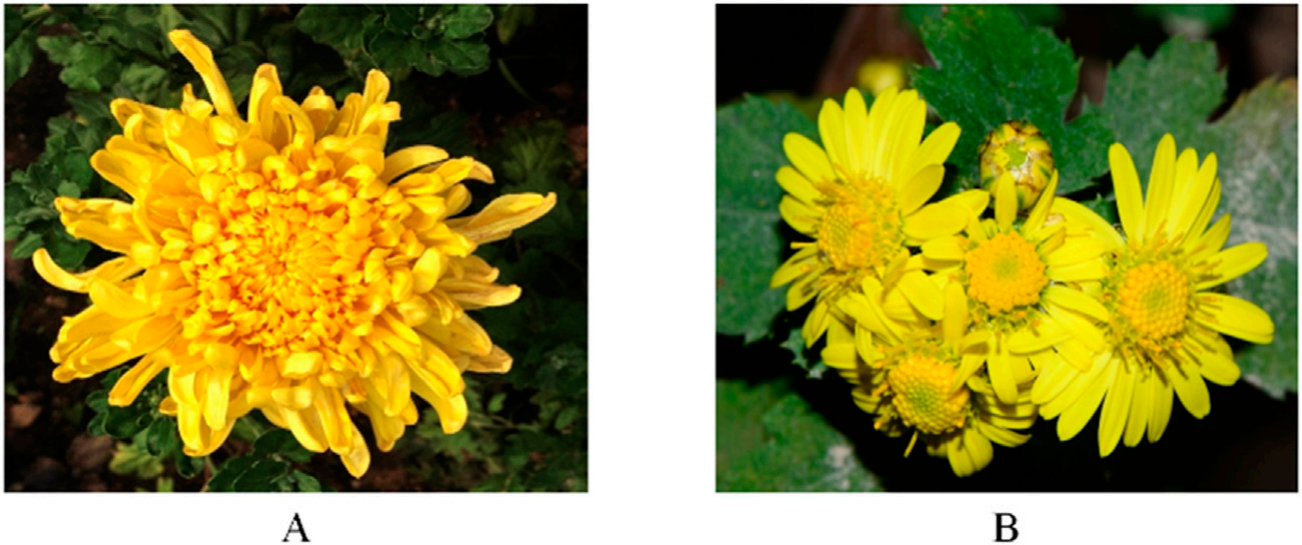
*Chrysanthemum morifolium* Ramat. and *Chrysanthemum indicum* Linné. **(A)** *Chrysanthemum morifolium* (CM) is the flower head of *Chrysanthemum morifolium* Ramat. **(B)** *Chrysanthemum indicum* (CI) is the flower head of *Chrysanthemum indicum* Linné. Two images are from Guangmin Li and Xinxin Zhu, respectively. Both images are from Plant Data Center of Chinese Academy of Sciences (https://www.plantplus.cn).

The application of CI (Yeju) in TCM can be traced back to the Qin and Han dynasties (Chen Caiying et al., 2015). The native range of CI includes China, the Eastern Himalayas, Inner Mongolia, Japan, South Korea, Nepal, *etc.* Additionally, it has a broad distribution across various Asian countries, as well as in several European and South American nations (Committee FoCE, 2011). This plant thrives in wide range of habitats, from grasslands on mountain slopes and thickets to wet areas near rivers, fields, roadsides, saline zones by seashores, and under scrub, typically at altitudes ranging from 100 to 2,900 m (Committee FoCE, 2011). The botanical morphological characteristics of CI are as follows: 1) Life form: Perennial herb. 2) Stem: Long or short procumbent rhizomes, with erect or diffuse stems that branch and are sparsely pilose; lower leaves senesce by anthesis. 3) Leaves: Middle stem leaves have petioles measuring 1–2 cm, with leaf blades ovate to long-ovate or elliptic-ovate, ranging from 3–7 (-10) × 2–4 (-7) cm. Both surfaces are pale green or olive-green, with the adaxial surface being sparsely pubescent and the abaxial surface less densely. The leaves are pinnatifid, pinnatilobed, or inconspicuously divided, with a truncate, somewhat cordate, or broadly cuneate base. 4) Flowers: The synflorescence is a lax terminal flat-topped cyme, bearing numerous or few capitula. Phyllaries are arranged in 5 rows, featuring broad scarious margins that are white or brown, with obtuse or rounded apices. Outer phyllaries are ovate or ovate-triangular, measuring 2.5–3 mm, while middle phyllaries are ovate at 6–8 mm, and inner phyllaries are narrowly elliptic, approximately 1.1 cm. Ray floret laminae are yellow, measuring 1–1.3 cm, with entire or 3-denticulate apices. 5) Fruits: Achenes measure 1.5–1.8 mm (Committee FoCE, 2011). (Figure 1).

### 2.2 Traditional uses

CM has a long history as a TCM. The earliest record of CM can be traced back to the *Shennong’s Herbal Classic*, which described its taste as bitter and neutral, and indicated its primary use for dizziness and pain caused by wind, and other conditions. The *Mingyi Bielu* noted CM tastes sweet and is non-toxic. According to *The Compendium of Materia Medica*, Juhua is widely believed to dispel wind-heat, benefit the liver, and tonify Yin”. A Qing Dynasty doctor, Xu Dachun, recorded that prolonged consumption of Juhua can tonify blood and Qi, enhance physical wellbeing, and promote longevity. Since Juhua can survive for a long time, there is a belief that consuming it can extend human life. ChP recorded that CM disperses wind and clears heat, pacifies the liver and brightens the eyes, and detoxifies. Clinically, it is used to treat wind-heat colds, headaches and dizziness, red and swollen eyes, diminished eyesight, sores, and abscess swelling (Commission CP, 2020). CM is extensively utilized in TCM, particularly as a primary ingredient in TCM prescription formulations such as Sangju Ganmao Wan, Huanglian Shangqing Wan, and Jinsang Kaiyin Wan. The 2020 edition of the ChP lists 1605 TCM formula and single-botanical drug preparations, among which 55 containing CM, accounting for approximately 3.4% of the total (Commission CP, 2020).

There are limited records of CI in ancient medical literature. The earliest record of CI can be traced back to the *Sheng Nong’s herbal classic* and it was recorded as “Jiehua” (Chen Caiying et al., 2015). In the *Bencao Qiuzhen*, CI is described as having the properties of “entering the lung and liver meridians”. The *Bencao Huiyan* noted: “CI has a bitter taste, is cold in nature, and slightly toxic. It functions to break up blood stasis, soothe the liver, and detoxify whitlow. Additionally, it can be used as a douche for scabies to dispel wind and kill parasites”. The 2020 edition of ChP recorded that CI has the functions of clearing heat, detoxifying, purging fire, and pacifying the liver, and clinically, it is used to treat conditions such as whitlow, sores, abscesses, and swelling, as well as symptoms like redness of the eyes, headache, and dizziness (Commission CP, 2020). The 2020 edition of ChP includes 16 TCM formula preparations containing CI (Commission CP, 2020). Table 2 provides details on the utilization of CM and CI as monarch medications.

**TABLE 2:** The preparations of CM and CI as monarch medicine were listed in ChP 2020 edition.

Name		Function
CM	Xiaoer Ganmao Keli (Cha, Koufuye)	Disperse wind and release the exterior, clear heat and detoxify
	Qiju Dihuang Wan (Koufuye, Pian)	Enrich the kidney and nourish the liver
	Xiongju Shangqing Wan (Pian, Shuiwan)	Clear heat and release the exterior, relieve pain
	Sangju Ganmao Wan (Pian, Heji)	Disperse wind and clear heat, diffuse the lung to suppress cough
	Qingre Yinhua Tangjiang	Clear heat and detoxify, diuresis
	Tianju Naoan Jiaonang	Pacify the liver to extinguish wind, activate blood and resolve stasis
	Mingmu Shangqing Pian	Clear heat and disperse wind, improve vision and relieve pain
	Shanju Jiangya Pian	Pacify the liver to subdue Yang
	Fuming Pian	Enrich the kidney and nourish the liver, tonify Yin and engender fluid, clear the liver to improve vision
	Xiaoer Tuirening Koufuye	Release the exterior and clear heat, resolve phlegm to suppress cough, detoxify to soothe the throat
CI	Yejuhua Shuan	Antibacterial and anti-inflammatory
	Xiasangju Keli	Clear the liver to improve vision, disperse wind and clear heat, relieve the dampness fixed impediment, detoxify the sore
	Biyan Qingdu Keli	Clear heat and detoxify, resolve phlegm and dissipate binds

## 3 Pharmacology

We conducted a comprehensive literature search in PubMed from 2000 to 2024 using the terms “Chrysanthemum morifolium”, “Chrysanthemum morifolium Ramat.”, “Chrysanthemum indicum”, “Chrysanthemum indicum Linné.”, as well as related keywords such as “Juhua”, “Yeju”, and “Yejuhua”. This search yielded a total of 785 articles (CM: n = 582, CI: n = 203.). After removing duplicates (n = 47) and review articles (n = 23), we carefully screened the titles and abstracts of the remaining articles and excluded those (n = 469) not relevant to the pharmacological effects of CM and CI. Ultimately, we included twenty-nine studies that explicitly examined the antimicrobial (n = 10), anti-inflammatory (n = 8), antioxidant (n = 7), and immunomodulatory (n = 4) effects of CM and CI, with a focus on their potential applications in infectious diseases.

**FIGURE 2:**
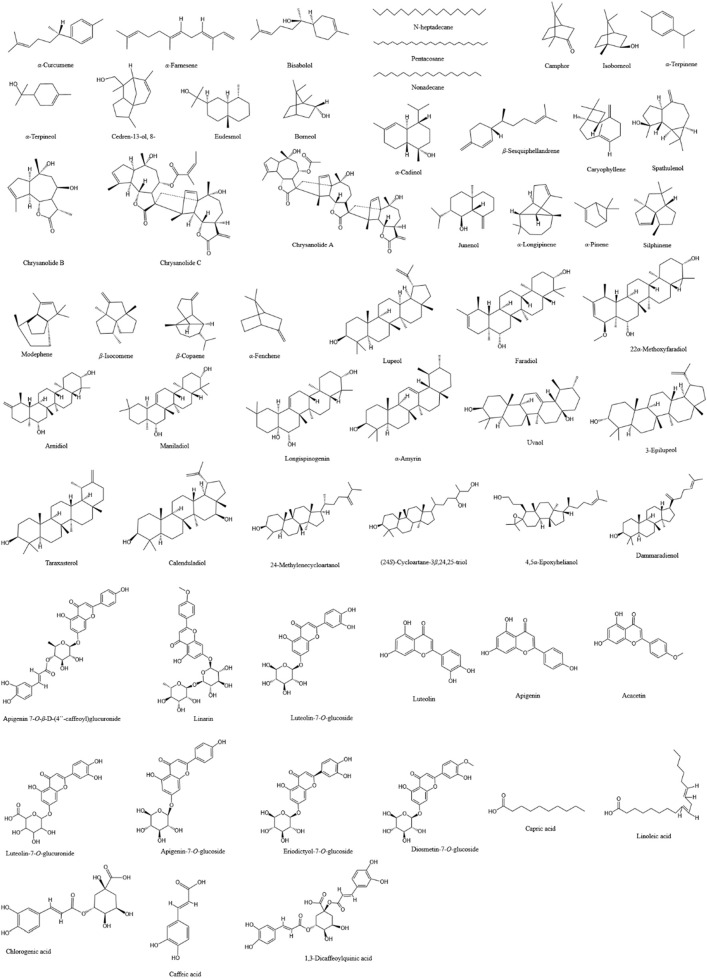
The chemical structures of the main compounds that may exert anti-infection-related pharmacological effects in CM and CI.

With the exploration of the potential applications of CM and CI in food, health products, and cosmetics, research into their chemical components has significantly expanded. They contain a rich array of compounds, including flavonoids, phenylpropanoids, terpenoids, triterpenoids, and others, which may endow them with anti-infective pharmacological effects such as antimicrobial, anti-inflammatory, and antioxidant properties. Our review of studies investigating the potential roles of CM and CI in combating infectious diseases revealed that they possess distinct advantages, which can be attributed to their different content and composition of specific compounds, particularly terpenoids and flavonoids. Consequently, we have summarized the major compounds analyzed in the included studies of CM and CI in Table 3 and illustrated these compounds in Figure 2.

**TABLE 3:** The primary compounds in CM and CI that are likely to exert anti-infection-related pharmacological effects.

No.	Compound	CM	CI	CM			CI		
				Plant part	Extraction	Ref.	Plant part	Extraction	Ref.
	Alkenes and terpenoids								
1	*α*-Curcumene	✓	✓	Flowers	HDE	Kuang et al. (2018), Liu et al. (2022), Youssef et al. (2020)	Flowers	HDE	Youssef et al. (2020)
2	*α*-Farnesene	✓		Flowers	HDE	Kuang et al. (2018)			
3	*β*-Bisabolene	✓		Flowers	HDE	Kuang et al. (2018)			
4	Bisabolol	✓		Flowers	HDE	Kuang et al. (2018)			
5	N-heptadecane	✓		Flowers	HDE	Kuang et al. (2018)			
6	Nonadecane	✓		Flowers	HDE	Kuang et al. (2018)			
7	N-pentacosane	✓	✓	Flowers	HDE	Kuang et al. (2018)	Flowers	HDE	Youssef et al. (2020)
8	Camphor	✓	✓	Flowers	HDE	Youssef et al. (2020)	Flowers	HDE	Youssef et al. (2020)
9	Isoborneol		✓				Flowers	HDE	Youssef et al. (2020)
10	*α*-Terpinene		✓				Flowers	HDE	Youssef et al. (2020)
11	Caryophyllene oxide	✓	✓	Flowers	HDE	Youssef et al. (2020)	Flowers	HDE	Youssef et al. (2020)
12	*α*-Terpineol	✓	✓	Flowers	HDE	Youssef et al. (2020)	Flowers	HDE	Youssef et al. (2020), Shunying et al. (2005)
13	Cedren-13-ol, 8-	✓	✓	Flowers	HDE	Youssef et al. (2020)	Flowers	HDE	Youssef et al. (2020)
14	*τ*-Eudesmol	✓		Flowers	HDE	Youssef et al. (2020)			
15	Borneol	✓		Flowers	HDE	Youssef et al. (2020)			
16	Chrysanolide A		✓				Flowers	EE	Gu et al. (2013)
17	Chrysanolide B		✓				Flowers	EE	Gu et al. (2013)
18	Chrysanolide C		✓				Flowers	EE	Gu et al. (2013)
19	*α*-Cadinol	✓		Flowers	HDE	Liu et al. (2022), Youssef et al. (2020)			
20	*β*-Sesquiphellandrene	✓		Flowers	HDE	Liu et al. (2022)			
21	Caryophyllene	✓		Leaves and stems	HDE	Liu et al. (2022)			
22	Spathulenol	✓		Flowers	HDE	Liu et al. (2022)			
23	Caryophyllen-5-ol	✓		Flowers	HDE	Liu et al. (2022)			
24	Junenol	✓		Flowers	HDE	Liu et al. (2022)			
25	*β*-Cadinol	✓		Flowers	HDE	Liu et al. (2022)			
26	Iso-caryophyllene	✓		Flowers	HDE	Liu et al. (2022)			
27	(*E*)-*β*-Farnesene	✓		Flowers	HDE	Liu et al. (2022)			
28	*α*-Longipinene	✓		Roots	EAE	Zhang et al. (2020)			
29	*α*-Pinene	✓		Roots	EAE	Zhang et al. (2020)			
30	*β*-Pinene	✓		Roots	EAE	Zhang et al. (2020)			
31	(*E*)-*β*-caryophyllene	✓		Roots	EAE	Zhang et al. (2020)			
32	Silphinene	✓		Roots	EAE	Zhang et al. (2020)			
33	Modephene	✓		Roots	EAE	Zhang et al. (2020)			
34	*α*-Isocomene	✓		Roots	EAE	Zhang et al. (2020)			
35	*β*-Isocomene	✓		Roots	EAE	Zhang et al. (2020)			
36	*β*-Copaene	✓		Roots	EAE	Zhang et al. (2020)			
37	*α*-Fenchene	✓		Roots	EAE	Zhang et al. (2020)			
38	Lupeol	✓	✓	Flowers	NSL of ME	Akihisa et al. (2005)	Flowers	70% EE	Kang et al. (2013)
39	Faradiol	✓		Flowers	NSL of ME	Akihisa et al. (2005)			
40	22*α*-Methoxyfaradiol	✓		Flowers	NSL of ME	Akihisa et al. (2005)			
41	Faradiol *α*-epoxide	✓		Flowers	NSL of ME	Akihisa et al. (2005)			
42	Taraxasterol	✓		Flowers	NSL of ME	Akihisa et al. (2005)			
43	Arnidiol	✓		Flowers	NSL of ME	Akihisa et al. (2005)			
44	Maniladiol	✓		Flowers	NSL of ME	Akihisa et al. (2005)			
45	Longispinogenin	✓		Flowers	NSL of ME	Akihisa et al. (2005)			
46	*α*-Amyrin	✓		Flowers	NSL of ME	Akihisa et al. (2005)			
47	Uvaol	✓		Flowers	NSL of ME	Akihisa et al. (2005)			
48	3-Epilupeol^a^	✓		Flowers	NSL of ME	Akihisa et al. (2005)			
49	Calenduladiol	✓		Flowers	NSL of ME	Akihisa et al. (2005)			
50	24-Methylenecycloartanol	✓		Flowers	NSL of ME	Akihisa et al. (2005)			
51	(24*S*)-Cycloartane-3*β*,24,25-triol	✓		Flowers	NSL of ME	Akihisa et al. (2005)			
52	4,5*α*-Epoxyhelianol	✓		Flowers	NSL of ME	Akihisa et al. (2005)			
53	Dammaradienol	✓		Flowers	NSL of ME	Akihisa et al. (2005)			
	Flavonoids								
54	Apigenin 7-*O*-*β*-D-(4″-caffeoyl)glucuronide	✓		Flowers	ME	Lee et al. (2003)			
55	Linarin	✓		Flowers	95% EE	Liu et al. (2020)			
56	Luteolin-7-*O*-glucoside	✓	✓	Flowers	95% EE; AE	Liu et al. (2020), Lee et al. (2021), Zhou et al. (2023)	Flowers	95% EBE	Cheng et al. (2005)
57	Luteolin	✓	✓	Flowers	95% EE; AE	Liu et al. (2020), Lii et al. (2010)	Flowers, leaves, and stems	ME; 95% EBE	Cheng et al. (2005), Yu et al. (2019)
58	Apigenin	✓		Flowers	95% EE; AE	Liu et al. (2020), Lii et al. (2010)			
59	Acacetin	✓		Flowers	EE	Liu et al. (2020)			
60	Luteolin-7-*O*-glucuronide	✓		Flowers	AE	Lee et al. (2021)			
61	Apigenin-7-*O*-glucoside	✓		Flowers	AE	Lee et al. (2021), Zhou et al. (2023)			
62	Eriodictyol-7-*O*-glucoside	✓		Flowers	AE	Zhou et al. (2023)			
63	Diosmetin-7-*O*-glucoside	✓		Flowers	AE	Zhou et al. (2023)			
64	Acacetin-7-rhamnoglucoside		✓				Flowers	95% EBE	Cheng et al. (2005)
	Phenylpropanoids								
65	Neochlorogenic acid	✓		Flowers	95% EE	Liu et al. (2020)			
66	Chlorogenic acid	✓	✓	Flowers	95% EE; AE	Liu et al. (2020), Lee et al. (2021)	Flowers, leaves, and stems	ME	Yu et al. (2019)
67	Caffeic acid	✓		Flowers	95% EE	Liu et al. (2020)			
68	Isochlorogenic acid C; 3,4-Dicaffeoylquinic acid	✓		Flowers	95% EE	Liu et al. (2020)			
69	Isochlorogenic acid A; 3,5-Dicaffeoylquinic acid	✓		Flowers	95% EE; AE	Liu et al. (2020), Lee et al. (2021)			
70	Isochlorogenic acid B 4,5-Dicaffeoylquinic acid	✓		Flowers	95% EE	Liu et al. (2020)			
71	1,3-Dicaffeoylquinic acid; 1,5-Dicaffeoylquinic acid	✓	✓	Flowers	95% EE; AE	Liu et al. (2020), Lee et al. (2021)	Flowers, leaves, and stems	ME	Yu et al. (2019)
	Others								
72	Capric acid	✓		Flowers	HDE	Kuang et al. (2018)			
73	Linoleic acid	✓		Flowers	HDE	Kuang et al. (2018)			
74	A New bisepoxylignan dendranlignan A	✓		Flowers	50% ACE	Zeng et al. (2020)			
75	Polysaccharide CMJA0S2	✓		Flowers	AE	Zheng et al. (2015)			
76	Polysaccharides	✓		Flowers	95% EE	Tao et al. (2018)			
77	Polysaccharides	✓		Flowers	95% EE	Tao et al. (2017)			
78	Polysaccharides	✓		Flowers	EE	Wang et al. (2022)			

HDE, Hydro distillation extraction; EE, ethanol extract; EAE, ethyl acetate extract; NSL of ME, NSL fraction of the methanol extract; ME, methanol extract; AE, aqueous extract; EBE, ethanol and butanol extract; ACE, acetone extract. a) Semisynthesized from compound lupeol (No.38).

### 3.1 Antimicrobial effects

CM and CI extracts exhibit potent antimicrobial effects, demonstrating potential to inhibit a wide range of microorganisms including bacteria, fungi, viruses, mycobacteria, and parasite and others (Kuang et al., 2018; Liu et al., 2022; Youssef et al., 2020; Shunying et al., 2005; Gu et al., 2013; Zhang et al., 2020; Akihisa et al., 2005; Lee et al., 2003). The differences in the types of inhibited microorganisms between CM and CI may be attributed to variations in their primary compounds, such as terpenoids and flavonoids.

#### 3.1.1 Antibacterial effect

Terpenoids have been reported to exhibit significant antibacterial activity by destroying the integrity of bacterial cell membranes or impairing essential bacteria functions (Yamaguchi, 2022; Meenu et al., 2023; Zacchino et al., 2017). Essential oils from CM and CI are rich in terpenoids (Liu et al., 2017; Peng et al., 2020; Shao et al., 2020; Wang Xl et al., 2006; Xue et al., 2018), which suggest that they possess potent antibacterial properties. For instance, the study conducted by Kuang et al. (Kuang et al., 2018) demonstrated that CM essential oil exhibits significant inhibitory effects against five bacterial strains: *Pseudomonas aeruginosa* (*P. aeruginosa*), *Salmonella enteritids* (*S. enteritidis*), *B. subtilis* (*Bacillus subtilis*), *Staphylococcus aureus* (*S. aureus*), and *Escherichia coli* (*E. coli*). The minimum inhibitory concentrations (MICs) were determined as follows: 0.33% for *P. aeruginosa*, 0.67% for both *S. enteritidis* and *B. subtilis*, and 1.30% for both *E. coli* and *S. aureus*. The main compounds isolated from CM essential oil are monoterpenes and sesquiterpenes, including *α*-curcumene (No.1), *α*-farnesene (No.2), *β*-bisabolene (No.3), bisabolol (No.4), n-heptadecane (No.5), nonadecane (No.6) and n-pentacosane (No.7) (Table 3). Notably, *α*-curcumene (No.1) is the most abundant compound among these compounds, accounting for 12.55% of the total composition (Kuang et al., 2018). These compounds are potentially associated with the antibacterial properties of CM essential oils. Furthermore, in a separate study, the content of *α*-curcumene (No.1) extracted from CI *via* hydro-distillation (1.23%) was significantly lower at 1.23% compared to 10.50% obtained from CM (Youssef et al., 2020). This suggests that CI may exhibit a weaker inhibitory effect on the aforementioned five bacteria.

Additionally, camphor (No.8), an oxygenated monoterpene, is the predominant compound in both CM (14.56%) and CI (36.69%) oils (Youssef et al., 2020). Notably, the essential oil of CI demonstrated superior antimicrobial activity against Gram-positive bacteria, including *B. subtilis*, *Streptococcus agalactiae*, and *Streptococcus pyogenes*, compared to the CM essential oil (Youssef et al., 2020). This enhanced activity may be attributed to the higher concentrations of camphor (No.8) and isoborneol (No.9) (7.64%) in CI (Youssef et al., 2020), which have been associated with antibacterial and antiviral properties (Chen et al., 2013; Astani et al., 2011; Costa et al., 2021). Therefore, in this study, essential oils rich in terpenoids demonstrated greater efficacy against Gram-positive bacteria than compared to Gram-negative bacteria (Youssef et al., 2020; Shunying et al., 2005). Moreover, the processed and air-dried flower oils of CI were evaluated against 15 microorganisms (Shunying et al., 2005). The processed flower oil exhibited the strongest antimicrobial activity against *Staphylococcus saprophyticus* (*S. saprophyticus*) (MIC: 0.78 mg/mL, minimum bactericidal concentration (MBC): 0.78 mg/mL), and the air-dried flower oil showed the most effective bactericidal activity against *E. coli* (MIC: 0.39 mg/mL, MBC: 0.39 mg/mL). The two oils possessed good inhibitory effects on *S. saprophyticus* (MIC: 1.56 mg/mL, MBC: 1.56 mg/mL) (Shunying et al., 2005). Furthermore, the air-dried essential oil showed superior bactericidal activity against *E*. *coli* (102 strains) at concentrations ranging from 3 to 56 μg/mL, likely due to its higher content of *α*-terpineol (No.12) at 3.32%, compared to 2.94% in the processed flower essential oil (Shunying et al., 2005). CM also contains *α*-terpineol (No.12), but at a lower concentration of only 0.65% (Youssef et al., 2020).

Furthermore, essential oils derived from different nonmedicinal parts of CM exhibit varying degrees of antibacterial activity. Specifically, Juhua demonstrates the most potent antibacterial effect against *S. aureus* (MIC: 10 mg/mL) compared to the stem and leaf extracts as well as root extracts of CM (Liu et al., 2022). The antibacterial efficacy of Juhua and stem and leaf extracts against *Propionibacterium acnes* (*P. acnes*) is comparable (MIC: 25 mg/mL), both of which are significantly higher than that of the root extract (Liu et al., 2022). This may be related to the high content of terpenoids in Juhua oil compared with the other two parts of essential oils. In this study, Juhua essential oil exhibited the highest terpenoid content among processed flowers at different stages (Jumi, Tianju, Juhua) and different parts of CM (Juhua, Stem and Leaf, Root), which likely contributes to its superior antibacterial efficacy. The composition of Juhua essential oil was predominantly oxygenated sesquiterpenes, with *α*-cadinol being the major constituent (28.62%, No.19). Notably, the content of heterospiroolefins, such as (*E*)-tibetin spiroether, decreased significantly from 44.81% in Jumi essential oil to 7.27% in Juhua essential oil. Despite this reduction, heterospiroolefins appear to have minimal impact on the antibacterial activity of CM (Liu et al., 2022). It is worth noting that heterospiroolefins were first isolated from CM, yet their specific antibacterial effects remain unclear.

#### 3.1.2 Antimycobacterial effect

Triterpenoids and essential oils extracted from CM and CI showed significant anti-mycobacterial activity against *Mycobacterium tuberculosis* (*M. tuberculosis*) (Youssef et al., 2020; Akihisa et al., 2005). For instance, Akihisa et al. (Akihisa et al., 2005) utilized the microplate Alamar Blue Assay (MABA) to identify twenty-nine 3-hydroxytriterpenoids from unsaponified lipid extracts of CM. Among these compounds, fourteen exhibited MIC values ranging from 4 to 64 μg/mL against *M. tuberculosis* (as detailed in Tables 3, 4). 3-Epilupeol (MIC: 4 μg/mL, No.48), derived from lupeol (No.38), and maniladiol (MIC: 4 μg/mL, No.44) showed the most potent anti-mycobacterial activity among the tested compounds (Akihisa et al., 2005). Notably, the half maximal inhibitory concentration (IC_50_) value of three-epilupeol exceeded 62.5 μg/mL, suggesting a selective toxicity towards *M. tuberculosis* (Akihisa et al., 2005). Although the antitubercular effect of CM and the extract was much lower compared to first-line antitubercular drugs, such as rifampin and isoniazid, these compounds still exhibited substantial activity against *M. tuberculosis* (Youssef et al., 2020; Akihisa et al., 2005).

**TABLE 4:** Detailed information of antimicrobial effects of CM and CI.

Extracts/compounds	Control	Mode	Detail	MIC/IC_50_/EC_50_/Active concentration	Ref.
CM					
Essential oil (Hydro distillation extraction)	Negative control: ethanol solution (95%)	-	Inhibits *P. aeruginosa*, *S. enteritidis*, *B. subtilis*, *S. aureus*, and *E. coli*	MIC = 0.33%, 0.67%, 0.67%, 1.30%, and 1.30%	Kuang et al. (2018)
Jumi, Tianju, Juhua, roots, and the stem and leaf essential oil (Hydro distillation extraction)	Positive control: Penicillin (*S. aureus*); erythromycin lactobionate (*P. acnes)*	*P. acnes/S. aureus*-induced THP-1 cells	Anti-bacterial: Inhibits *P. acnes* and *S. aureus*	Jumi: MIC = 25 and 10 mg/mL; Tianju: MIC = 25 and 10 mg/mL; Juhua: MIC = 25 and 10 mg/mL; stem and leaf: MIC = 25 and 20 mg/mL; roots: MIC = 50 and 50 mg/mL Control: penicillin: *S. aureus*:MIC = 0.0035 mg/mL; erythromycin lactobionate: *P. acnes*: MIC = 0.061 mg/mL. The MIC of the remaining bacteria was not detected Juhua and stem and leaf: IL-1β in *P. acnes*-induced THP-1 cells↓	Liu et al. (2022)
Twenty-nine 3-hydroxytriterpenoids (NSL fraction of the methanol extract)	Positive control: Rifampin	Vero cells	Anti-tuberculosis activity agaist *M. tuberculosis* strain H37Rv	1)3-epilupeol: MIC = 4 μg/mL, IC_50_ > 62.5 μg/mL 2)Compounds 39–47, 49–53: MIC = 4–64 μg/mL 3)Others: MIC >64 μg/mL	Akihisa et al. (2005)
Apigenin 7-*O*-*β*-D-(4″-caffeoyl) Glucuronide (Methanol extract)	Positive control: Apigenin 7-*O*-*β*-D-glucuronide and L-chicoric acid	HIV-1_IIIB_ infected MT-4 cells	Antiviral: Inhibits HIV and HIV-1 integrase activity	IC_50_ = 7.2 ± 3.4 μg/mL, EC_50_ = 41.86 ± 1.43 μg/mL	Lee et al. (2003)
12 cultivars of CM roots extracts (Ethyl acetate extract)	Negative control: Organic solvent ethyl acetate	-	Antifungal: “Xiao Huang Ju” was the only cultivar that exhibited significant inhibitory effects on all three species of *Magnaporthe oryzae*, *Verticillium dahliae*, and *Fusarium oxysporum* (*p* < 0.05)	-	Zhang et al. (2020)
CI					
Essential oils of air-dried and processed flowers (Hydro distillation extraction)	Positive control: Levofloxacin	-	Antimicrobial activity against 15 microorganisms *Bacillus subtilis*, *Staphylococcus aureus*, *Staphylococcus aureus*, *Proteus vulgaris*, *Salmonella typhi*, *Saccharomyces cerevisiae*, *Hansenula anomala, Candida* sp., *Klcbsiella pneumoniae*, *Citrobacter freundill*, *Enterobacter cloacae*, *Escherichia coli*, *Staphylococcus saprophyticus*, *Enterococcus faecalis*, and *Proteus mirabilis*	A concentration of 3.00 mg/disc Air-dried: MIC = 3.13, 3.13, 3.13, 12.50, 6.25, 12.50, 6.25, 3.13, 6.25, 25.00, 6.25, 0.39, 1.56, 25.00, and 50.00 mg/mL Processed: MIC = 3.13, 6.25, 3.13, 50.00, 12.50, 12.50, 1.56, 0.39, 6.25, 25.00, 6.25, 6.25, 0.78, 3.13, and 25.00 mg/mL Control: MIC = 0.61, 0.61, 0.61, 0.61, 1.22, nt, nt, nt, 0.31, 39.06, 4.88, 2.44, 9.77, 9.77, and 39.06 mg/mL	Shunying et al. (2005)
Chrysanolide B, Chrysanolide C, Chrysanolide A (ethanol extract)	Positive control: Lamivudine	HepG 2.2.15 cell line	Antiviral: Inhibits the secretion of HBsAg and HBeAg	HBsAg: IC_50_ = 131.28, 33.91, and 6.67 μM; HBeAg: IC_50_ = 144.48, 30.09, and 6.23 μM	Gu et al. (2013)
70% ethanol extract	Blank control: DMSO	Parental BL41 cells, their LMP1 expressing counterparts, and LCL	Antiviral:1)Inhibits LMP1 CTAR 1 and 2-induced NF-κB activation possibly by interfering with IKK*α* and IKK*β* activation 2)Reduces the viability of EBV-transformed LCL viability by inducing apoptosis	Concentration: 1, 2, 3, and 4 μg/mL (in a dose- and time-dependent manner)	Kim et al. (2012)
1)CH_2_Cl_2_ fraction of CI 2)lupeol (80% ethanol extract)	1)DMSO 2)HEK293 cells were co-transfected with Renilla luciferase plasmids	1)LCLs, HFF, HeLa and BL41 cells 2)HEK293 cells were co-transfected with pSG5 or pSG5-FLAG-LMP1 plus NF-κB dependent firefly luciferase	Antiviral: Attenuates LMP1-induced NF-κB activation and LCL viability	1)CH_2_Cl_2_ fraction: LCLs: Active concentration: 100 μg/mL; IC_50_ at 24, 48 and 72 h were 97.3, 55.8 and 45.2 mM, respectively. HFF: IC_50_ at 72 h was 145.5 mM. HeLa: IC_50_ at 48 and 72 h were 84.3 and 109.7 mM BL41: IC_50_ at 24, 48 and 72 h were 150.9, 93.7 and 91.4 mM. Other data were not determined 2)Lupeol: Active concentration: 50 μg/mL; IC_50_ at 24, 48 and 72 h were 109.9, 57.6 and 51.8 mM, respectively	Kang et al. (2013)
CM &CI					
Essential oils (Hydro distillation extraction)	Positive control: acyclovir	Vero cells	Antiviral: Inhibits VSV, HAV, and HSV-1	CI: IC_50_ = 3.14, 3.38, and 3.51 μg/mL. CM: IC_50_ = 3.69, 3.80, and 3.73 μg/mL (Control: IC_50_ = 2.21, 1.84, and 1.49 μg/mL) (*p* < 0.05)	Youssef et al. (2020)
	Positive control: Ampicillin (Gram-positive bacteria); gentamycin (Gram-negative bacteria); clotrimazole (fungi)	-	Antimicrobial: Gram-positive bacteria *Bacillus subtilis, Streptococcus agalactiae and Streptococcus pyogenes*	CI was more effective than CM. CI: MIC = 62.5 μg/mL. CM and CI exerted weak activity *versus* the examined Gram-negative bacteria and fungal strains with MICs >500 μg/mL	Youssef et al. (2020)
	Positive control: Isoniazid	-	Anti-mycobacterial activity against *M. tuberculosis*	CM and CI: IC_50_ = 7.36 and 6.73 μg/mL (Control: IC_50_ = 0.038 μg/mL) (*p* < 0.05)	Youssef et al. (2020)
	Positive control: Clarithromycin	-	Anti-Helicobacter pylori	CM and CI: IC_50_ = 3.78 and 3.63 μg/mL (Control: IC_50_ = 0.76 μg/mL) (*p* < 0.05)	Youssef et al. (2020)
	Positive control: Diminazene	-	Antiparasitic: Anti-trypanosomal activity	CM and CI: IC_50_ = 49.02 and 45.89 μg/mL (Control: IC_50_ = 0.075 μg/mL) (*p* < 0.05)	Youssef et al. (2020)

“-”: Not mentioned. “nt”: Not tested. Concentration for 50% of maximal effect (EC50), C-terminal activation regions (CTAR).

#### 3.1.3 Antifungal effect

A study evaluating the antifungal activities of extracts from 12 cultivars of CM roots against *Magnaporthe oryzae*, *Verticillium dahliae*, and *Fusarium oxysporum* revealed that only “Xiao Huang Ju” exhibited significant inhibitory effects on all tested fungi (Zhang et al., 2020). Table 3 summarizes the key compounds identified in the 12 cultivars through principal component analysis. However, a separate study reported that both CM and CI oils demonstrated weak antifungal activity against *Aspergillus fumigatus*, *Candida albicans*, *Geotrichum candidum*, and *Syncephalastrum racemosum*, with MIC values exceeding 500 μg/mL (Youssef et al., 2020).

#### 3.1.4 Antiviral effect

Both CM and CI possess antiviral properties (Youssef et al., 2020; Gu et al., 2013; Kang et al., 2013; Lee et al., 2003; Kim et al., 2012). For instance, the flavonoid compound apigenin 7-*O*-*β*-D-(4″-caffeoyl)glucuronide (No.54), isolated from CM, exhibited potent HIV-1 integrase inhibitory activity and anti-HIV effects in HIV-1_IIIB_-infected MT-4 cells (Lee et al., 2003). Moreover, a unique sesquiterpenoid trimer (Chrysanolide A, No.16), along with its biogenetically related monomer (Chrysanolide B, No.17) and dimer (Chrysanolide C, No.18), isolated from CI, exhibited strong inhibitory activity against the secretion of HBsAg and HBeAg (Gu et al., 2013). Additionally, both CM and CI essential oils showed antiviral activity against vesicular stomatitis virus (VSV), hepatitis A (HAV) and herpes simplex type-1 (HSV-1) (Youssef et al., 2020). Notably, the antiviral activity of CI was dose-dependent, with VSV being the most sensitive to CI’s antiviral effects (Youssef et al., 2020).

Epstein-Barr virus (EBV) latent infection membrane protein 1 (LMP1) plays a critical role in EBV-mediated B lymphocyte transformation, with LMP1-induced nuclear factor kappa-B (NF-κB) activation being crucial for the survival of lymphoblastoid cell lines (LCLs). CI exhibited a potent inhibitory effect on EBV LMP1-induced NF-κB activation and significantly reduced the viability of EBV-transformed LCLs in a dose- and time-dependent manner (Kim et al., 2012). This inhibition likely involves blocking LMP1-induced IKK*α* and IKK*β* activation, without affecting the viability of human foreskin fibroblasts, EBV-negative Burkitt lymphoma cells, or HeLa cells (Kim et al., 2012). Furthermore, both the CH2Cl2 fraction of CI and lupeol (No.38) significantly attenuated LMP1-induced NF-κB activation and reduced the viability of LCLs. Notably, the CH_2_Cl_2_ fraction of CI exhibited superior antiviral properties compared to lupeol. Additionally, lupeol may synergistically interact with unidentified compounds in the CH_2_Cl_2_ fraction of CI to further diminish LMP1-induced NF-κB activation and LCL viability (Kang et al., 2013).

#### 3.1.5 Other effects

CM and CI have also exhibited potential antimicrobial effects against various microorganisms, including *Helicobacter pylori* and trypanosomes (Youssef et al., 2020). For example, the essential oils of CM and CI showed an anti-*H. pylori* activity with IC_50_ values of 3.63 μg/mL and 3.78 μg/mL, respectively, which are comparable to those of clarithromycin (Youssef et al., 2020).

The antimicrobial activity of CM and CI is strongly correlated with their terpenoid and triterpenoid content. Terpenes enhance lipophilicity and membrane permeability, leading to significant disruption of oxidative phosphorylation and electron transport chains, thereby severely impairing energy production and inducing autooxidation and peroxidation, ultimately resulting in bacterial lysis (Gamal El-Din et al., 2018). The potency of their antimicrobial effects is also influenced by the concentration of key compounds such as *α*-curcumene (No.1), camphor (No.8), and *α*-terpineol (No.12). Studies have demonstrated that while CI and CM exhibit weak antifungal activity, CM shows superior antifungal efficacy when different parts of the extract are used. Furthermore, CI demonstrates a stronger inhibitory effect on specific viruses. Additionally, some studies report antimicrobial activity using only IC_50_ or MIC values, but not both, which may introduce bias in their results. More details are provided in Table 4. Although the antimicrobial activity of CM and CI has been extensively studied, the detailed mechanism of their antimicrobial action has not been reported.

### 3.2 Anti-inflammatory effects

When an infection occurs, the immune system initiates an inflammatory response to combat invading pathogens (Zhang and Wang, 2014). However, excessive or prolonged inflammation can cause tissue damage and exacerbate disease severity. In severe cases, uncontrolled inflammation may lead to sepsis, widespread tissue injury, and organ failure (Esposito et al., 2017). Reducing inflammation can mitigate pathogen-induced tissue damage and bodily discomfort. Studies have shown that both CM and CI extracts exhibit significant anti-inflammatory effects by modulating inflammation-related pathways (Liu et al., 2020; Lee et al., 2021; Zhou et al., 2023; Yu et al., 2019; Zeng et al., 2020; Wu et al., 2014; Lee et al., 2009; Cheon et al., 2009).

CM and CI extracts demonstrated potential efficacy in treating acute lung injury (ALI). In a mouse model of lipopolysaccharide (LPS)-induced, both extracts significantly alleviated lung histopathological damage, reduced the wet-to-dry lung weight ratio and lung injury score, and were associated with decreased levels of pro-inflammatory cytokines, including IL-6 and tumor necrosis factor (TNF)-*α* (Liu et al., 2020; Wu et al., 2014). CM extract can attenuate the production of pro-inflammatory cytokines (such as IL-6 and TNF-α), enhance the secretion of anti-inflammatory cytokines (including transforming growth factor-*β*1 (TGF-*β*1) and IL-10), and mitigate the oxidative stress by increasing total antioxidant capacity (TAC) and reducing malondialdehyde (MDA) levels in mice with ALI. However, the precise mechanisms underlying these effects remain to be elucidated (Liu et al., 2020). The therapeutic efficacy of CI may be attributed to its ability to downregulate both the Toll-like receptor 4 (TLR4) and MyD88-dependent NF-κB signaling pathways (Wu et al., 2014).

The other TLR4 signaling pathway is MyD88-independent and TRIF-dependent, leading to the phosphorylation and nuclear translocation of IRF3. A newly discovered compound, bisepoxylignan dendranlignan A (BDA), isolated from CM, has been shown to reduce the production of inflammatory cytokines TNF-α, IL-2, and interferon (IFN)-*γ* in LPS-stimulated H9c2 cells (Zeng et al., 2020). BDA significantly decreased the nuclear translocation and phosphorylation levels of IRF3, the NF-κB heterodimer component p65, and one of the AP-1 components, c-Jun. However, it did not significantly affect the protein expression of TLR4, MyD88, or TRIF, suggesting that BDA inhibits TLR4 signaling downstream of these proteins (Zeng et al., 2020). Additionally, molecular docking studies revealed that BDA can occupy the ligand-binding site of the TLR4-MD2 complex, indicating that it may inhibit inflammation by blocking the TLR4 signaling pathway (Zeng et al., 2020).

Hyaluronidase (HAase) is an endoglycosidase important for the metabolism of hyaluronic acid (HA), a linear acidic mucopolysaccharide. HAase plays a significant role in inflammation by enhancing the production of cytokines IL-1β and TNF-α by macrophages, as well as their allostimulatory capacity (Sudha and Rose, 2014; Jiang et al., 2011; Termeer et al., 2000). Studies have found that inhibiting HAase can prevent HA degradation, thereby mitigating inflammatory responses (Shibata et al., 2002). For example, in an *in vitro* inflammatory model, Zhou et al. (2023) reported that CM extract dose-dependently inhibited HAase activity. Further screening identified four compounds 56, 61, 62 and 63 as the key inhibitors, and these compounds significantly reduced the production of inflammatory mediators nitric oxide (NO) and IL-6, and suppressed the mRNA expression of inducible NO synthase (iNOS) and IL-1β in both mouse and human macrophages.

In addition, the anti-inflammatory mechanism of CI may be associated with the regulation of apoptosis-associated speck-like protein (ASC) phosphorylation and the MAPKs and NF-κB-dependent signaling pathways (Yu et al., 2019; Cheon et al., 2009). For instance, *in vivo* studies have demonstrated that CI can inhibit the recruitment of total cells and Ly6G^+^/F4/80^-^ neutrophils as well as reduce the secretion of inflammatory cytokines in peritonitis mice. *In vitro,* CI has been shown to inhibit the activation of the nucleotide-binding oligomerization domain (NOD)-like receptor (NLR)3 and the HIN-200 family member absent in melanoma 2 (AIM2) inflammasomes, leading to decreased production of IL-1β and caspase-1 (Yu et al., 2019). The phosphorylation of ASC regulates the activity of inflammasomes such as NLR3 and AIM2 inflammasomes through the formation of ASC specks (Hara et al., 2013). In studies, treatment with CI resulted in reduced translocation formation and inhibited phosphorylation, while JNK phosphorylation was not implicated in this pathway (Yu et al., 2019). Given that JNK phosphorylation is recognized as an upstream regulator of ASC phosphorylation (Hara et al., 2013), the observed effects may be associated with the modulation of ASC phosphorylation. However, the specific pathway through which this occurs remains to be elucidated.

LPS can activate a signaling pathway involving mitogen-activated protein kinases (MAPKs), including ERK1/2, JNK1/2 and p38MAPK (Moens et al., 2013). MAPKs inhibitors have been shown to suppress the regulation of iNOS and cyclooxygenase (COX)-2 genes (Kim and Kim, 2005). Overexpression of iNOS and COX-2 leads to NF-κB activation, resulting in increased production of nitric oxide (NO) and prostaglandin E_2_ (PGE_2_), which exacerbates inflammatory responses (D'Acquisto et al., 1997). CI extract significantly inhibited the LPS-induced production of inflammatory mediators NO and PGE_2_, as well as inflammatory cytokines TNF-α and IL-1β, in RAW 264.7 macrophages in a dose-dependent manner (Cheon et al., 2009). Additionally, CI extract suppressed the mRNA and protein expression of iNOS and COX-2. Further studies revealed that CI can inhibit the nuclear translocation of NF-κB p65 subunits by preventing IκB*α* phosphorylation and also inhibit the phosphorylation of ERK and JNK, suggesting that the anti-inflammatory effects of CI are mediated through both MAPK and NF-κB pathways (Cheon et al., 2009). More details are provided in Table 5.

**TABLE 5:** Detailed information of anti-inflammatory effects of CM and CI.

Extracts/compounds	Active concentration/dose	Vitro/vivo	Mode	Detail	Mechanism	Ref.
CM						
95% ethanol extract	50, 100, and 200 mg/kg for 7 days	Vivo	LPS-induced ALI in mice	1)Lung histopathological injury and the ratio of wet/dry lung weight and lung index↓; the number of white blood cells, lymphocytes and neutrophils↓ 2)TNF-α and IL-6 production↓; TGF-*β*1 and IL-10 production↑ 3)TAC activity↑; MDA contents↓	-	Liu et al. (2020)
Hot water extract	2 and 4 g/kg for 13 weeks	Vivo	45% HF rats	1)In serum: TC, TG, and HDL-C↓ 2)In epididymal adipose tissue: Gene expression involved in adipogenesis↓, pro-inflammation (TNF-α, IL-6, and MCP1 mRNA levels of in eWAT)↓; the M1 macrophage phenotype↓; GPDH and NF-κB activities↓ 3)In the liver: AMPK activity↑: Hepatic fat accumulation↓, gene expression related to fat synthesis↓ and oxidation↓ 4)Muscle mitochondrial size↑; mtDNA content↑; SIRT1, PGC-1*α*, and PGC-1*α*-target genes expression↑	AMPK-SIRT1 pathway	Lee et al. (2021)
1)Aqueous extract 2)Compound 89, 71, 65 and 67	1)1.00, 2.00, and 4.00 mg/mL 2)1,000 μM	Vitro	1)LPS-induced RAW 264.7 and THP1 cells 2)Murine and human macrophages	1)Showed 8.31, 24.25, and 66.51% inhibition of HAase, respectively 2)Showed 40.15, 44.85, 18.04, and 24.15% inhibition of HAase, respectively; iNOS and IL-1β mRNA expression↓, and NO and IL-6 production↓	-	Zhou et al. (2023)
Bisepoxylignan dendranlignan A (50% acetone extract)	1.97 μM	Vitro	LPS-stimulated H9c2 cells	1) TNF-α, IL-2 and IFN-γ production↓ 2)The nuclear localization and the levels of phosphorylated of c-JUN, p-P65 and p-IRF3↓	TLR4 signaling pathway	Zeng et al. (2020)
CI						
Supercritical carbon dioxide fluid extract	40, 80 and 120 mg/kg for 7 days	Vivo	LPS-induced ALI mice	1)Lung histopathological injury↓ 2)TNF-α, IL-1β, and IL-6 production↓ 3)MPO and MDA levels↓; SOD, CAT, amd GPX activities↑ 4)The NF-κB activation and TLR4/MyD88 expression↓	TLR4/MyD88-dependent NF-κB signaling pathway	Wu et al. (2014)
70% ethanol extract	200 mg/kg for 10 days	Vivo	TPA-induced dermatitis mice	1)Local ear edema, skin thickness and tissue weight↓ 2)TNF-α and IL-*β* production↓ 3)Neutrophil-mediated MPO activity↓	-	Lee et al. (2009)
Methanol extract	1)100 μg/mL 2)intraperitoneal: 50 and 100 mg/kg; oral: 200 g/kg	1)Vitro 2)Vivo	1)LPS-primed BMDMs 2)MSU-induced murine peritonitis model	1)*In vitro*: NLRP3 and AIM2 inflammasomes activation↓ ASC speck formation and translocation↓, caspase-1 and IL-1β production↓; ASC phosphorylation↓ and no changes of JNK phosphorylation 2)*In vivo*: The recruitment of MSU-induced total cells and Ly6G^+^/F4/80^−^neutrophils in peritonium↓; IL-1β production↓	ASC phosphorylation independently of JNK phosphorylation	Yu et al. (2019)
70% ethanol extract	25, 50, 100 and 200 μg/mL	Vitro	LPS-induced RAW 264.7 macrophages	1)NO, PGE_2_, TNF-α, and IL-1β production↓ 2)mRNA and protein expression of iNOS and COX-2↓ 3)Nuclear translocation of NF-κB p65 subunits↓ and IκB*α* phosphorylation↓ 4)Phosphorylation of ERK, JNK, and p38. (only at 200 μg/mL)↓	MAPKs and NF-κB-dependent pathways	Cheon et al. (2009)

“↓”: Reduce or downregulate. “↑”: Increase or upregulate. “-”: not mentioned.

HF, high-fat; TC, total cholesterol; TG, triglyceride; HDL-C, high-density lipoprotein cholesterol; GPDH, glycerol-3-phosphate dehydrogenase; mtDNA, mitochondrial DNA; TPA, 12-O-tetradecanoyl-phorbol-13-acetate; SOD, superoxide dismutase; CAT, catalase; GPX, glutathione peroxidase; MSU, monosodium urate; BMDMs, bone marrow-derived macrophages.

### 3.3 Antioxidant effects

Treating oxidative imbalance is crucial in the management of infectious diseases (Ashique et al., 2025). Reactive oxygen species (ROS), which are by-products of cellular metabolism, exhibit a dual nature: they are beneficial to cells at low concentrations but become detrimental at high levels (Sander et al., 2022). For instance, ROS contribute to pathogen destruction; however, their excessive accumulation can induce damage to cellular components such as lipids, proteins, and DNA (Sander et al., 2022). Moreover, overproduction of ROS leads to oxidative stress, resulting in bodily damage, various disease states, impaired immune function, and exacerbated inflammatory responses. This creates a vicious cycle that hinders recovery (Ashique et al., 2025). In contrast, inflammation can result in the recruitment of intravascular neutrophils to the alveolar space and lung parenchyma (Looney et al., 2006), leading to the subsequent release of proteases and generation of ROS. Moreover, ROS are closely associated with lipid peroxidation products such as myeloperoxidase (MPO) and MDA, as well as the modulation of antioxidant enzyme activities, including SOD, CAT, and GPX (Looney et al., 2006). Antioxidants can neutralize ROS, thereby protecting cells from oxidative damage and enhancing the immune system’s ability to combat infections effectively. Essential oils derived from CM and CI exhibit potential as natural preservatives due to their antioxidant properties (Lii et al., 2010; Zheng et al., 2015; Zhan et al., 2022; Hao et al., 2021; Lin et al., 2010; Tian et al., 2019; Zhang et al., 2019; Kim et al., 2017).

CM extract and its flavonoids, apigenin and luteolin, exhibit potent antioxidant properties by resisting oxidative stress (Lii et al., 2010; Zhan et al., 2022; Hao et al., 2021). They enhance the levels of antioxidant enzymes such as SOD, CAT, and GPX, upregulate the expression of the antioxidant gene heme oxygenase-1 (HO-1), and decrease the production of reactive oxygen species (ROS), MDA, MPO, and 2,2-diphenyl-1-picrylhydrazyl (DPPH) radicals (Lii et al., 2010; Zheng et al., 2015; Zhan et al., 2022; Hao et al., 2021; Lin et al., 2010; Tian et al., 2019). Moreover, CM can inhibit cell apoptosis, modulate cell cycle progression, and reduce the expression of pro-apoptotic proteins including Bax, cleaved caspase-3, and cleaved poly (ADP-ribose) polymerase (PARP) (Zheng et al., 2015; Hao et al., 2021; Tian et al., 2019). Further studies suggest that these effects may be mediated through several signaling pathways, such as arginine and purine metabolism, phosphatidylinositol 3-kinase (PI3K)/Akt, PI3K/Akt-mediated nuclear factor erythroid 2-related factor 2 (Nrf2)/HO-1, and Nrf2 signaling pathways (Lii et al., 2010; Zhan et al., 2022; Hao et al., 2021; Tian et al., 2019).

CI also exhibits comparable antioxidant effects to CM, such as significantly enhancing the activities of antioxidant enzymes (Zhang et al., 2019; Kim et al., 2017). In addition, CI could not only inhibit the increased Bax/Bcl-2 ratio and activation of cleaved caspase-3 in the liver and brain but also reduce the levels of inflammatory cytokines, including IL-1β, IL-6, and TNF-α (Zhang et al., 2019). Furthermore, studies have found that both CM and CI exhibit peak antioxidant capacity when heated at 100°C for 45 min (Yu et al., 2023). Detailed information is provided in Table 6. Moreover, the anti-inflammatory effects of CM and CI are discussed in Section 3.2, while their antioxidant properties are summarized in Table 5 (Liu et al., 2020; Lee et al., 2021; Wu et al., 2014; Lee et al., 2009).

**TABLE 6:** Detailed information of antioxidative effects of CM and CI.

Extracts/compounds	Active concentration/dose	Vitro/vivo	Mode	Detail	Mechanism	Ref.
CM						
Aqueous extract	800, 1,600 and 2,400 μg/mL	Vitro	H_2_O_2_-treated L-O_2_ cells	1)H_2_O_2_-induced cell death↓ 2)SOD, CAT and GPX activities↑; MDA↓, ROS content↓ and mitochondrial membrane potential↑ 3)L-arginosuccinate, citrulline and inositol monophosphate↑. (*p* < 0.05)	Arginine biosynthesis and IMP synthesis	Zhan et al. (2022)
Aqueous extract	20, 60, and 100 μg/mL	Vitro	Human retinal pigment epithelial cell line (ARPE-19 cells)	1)ROS production↓. (in dose-dependent) 2)Proapoptotic related protein expression: cleaved caspase-3, cleaved PARP, and Bax/Bcl-2 ratio↓. (in dose-dependent) 3)Catalase, GCLc, SOD2, and NQO-1 expression↑. (100 μg/mL) 4)Akt phosphorylation↑(100 μg/mL); Nrf2 nuclear translocation and its downstream HO-1↑. (in a dose-dependent manner) (These protective effects can be reversed by a PI3K inhibitor. (LY294002)) (*p* < 0.05)	PI3K/Akt-mediated Nrf2/HO-1 signaling pathway	Hao et al. (2021)
1)Aqueous extract 2)95% ethanol extract 3)Flavonoids: apigenin (No.58) and luteolin (No.57)	1)100, 250 and 1,000 μg/mL 2)25, 100 and 250 μg/mL 3)Flavonoids: 20 and 50 μM	Vitro	oxLDL-induced HUVEC and HL-60 cells	1)ROS production in dose-dependent↓. (250 and 1,000 μg/mL; 100 and 250 μg/mL; 50 μM; 20 and 50 μM) 2)Dephosphorylation of Akt and CREB in a dose-dependent manner↓; the expression of ICAM-1 and E-selectin and adhesion of HL-60↓. (250 μg/mL; 250 μg/mL; 50 μM; 50 μM) (These protective effects can be reversed by a PI3K/Akt signaling pathway inhibitor (Wortmannin)). (*p* < 0.05)	PI3K/Akt signaling pathway	Lii et al. (2010)
A water-soluble polysaccharide (CMJA0S2) (Boiling-water extraction)	Active concentration:0.1 mg/mL	Vitro	H_2_O_2_-induced PC12 cells	1)Scavenging rate with DPPH in concentration-dependent↑. (0.1–0.8 mg/mL) 2)cell viability↑	-	Zheng et al. (2015)
70% ethanol extract	1)Vivo: 110, 220 and 440 mg/kg for 8 days 2)Vitro: 50, 100 and 200 μg/mL	Vivo and vitro	1)Alcohol-, CCl_4_-induced liver injury rats 2)APAP-treated HL-7702 cells	*In vivo*: 1)Liver index↓ and serum ALT and AST↓. 2)In serum: MDA content↓ and SOD activity↑ *In vitro*:1)ALT and AST↓. 2)Cell viability↑ and cell morphological deterioration↓(in a dose-dependent manner). 3)ROS production↓, SOD activity↓ and GSH content↓ (in a dose-dependent manner). 4)Bcl-2, Bax and Caspase-3 expression↓. 5)Nuclear translocation and the expression of Nrf2 as well as its downstream gene HO-1↑. (These antioxidant effects can be reversed by Nrf2 siRNA) (*p* < 0.05)	Nrf2 signaling pathway	Tian et al. (2019)
CI						
Supercritical carbon dioxide fluid extract	100, 150 and 300 mg/kg	Vivo	D-galactose-induced hepatic and cerebral injury mice	1)Body weight↑, the decline of thymus and spleen indexes↑, and ALT and AST levels↓ 2)In the liver and brain: SOD, CAT, and GPX activities↑; MDA↓. IL-1β, IL-6, and TNF-α production↓. The increase of Bax/Bcl-2 ratio↓ and cleaved caspase-3 activation↓. (*p* < 0.05)	-	Zhang et al. (2019)
70% ethanol extract	200 mg/kg	Vivo	Male Mongolian gerbils underwent ischemia surgery	In CA1 pyramidal cells: SOD1, CAT and GPX immunoreactivities↑. (*p* < 0.05)	-	Kim et al. (2017)

“↓”: Reduce or downregulate. “↑”: Increase or upregulate. “-”: not mentioned.

H_2_O_2,_ Hydrogen peroxide; GCLc, glutamate-cysteine ligase catalytic subunit; NQO1, NAD(P)H:quinone xidoreductase 1; ALT, alanine transaminase; AST, aspartate aminotransferase; siRNA, small interfering RNA; ox, oxidized; HUVEC, human umbilical vein endothelial cells.

### 3.4 Immunomodulatory effects

The immune system responds to exogenous factors encountered by the body and employs defense mechanisms to counteract these challenges. For instance, LPS, a major component of the outer membrane of Gram-negative bacteria, can bind to TLR4 and activate the MyD88-mediated NF-κB signaling pathway, ultimately leading to inflammatory responses (Hamad et al., 2023). Consequently, LPS is frequently utilized to induce inflammatory models in experimental settings. Moreover, abnormal immune responses are prevalent in infectious diseases such as influenza and COVID-19 (Han et al., 2018; Chen et al., 2024). Furthermore, with the increasing prevalence of antibiotic resistance, immunomodulatory agents have emerged as promising alternatives for treating infectious diseases (Yang et al., 2017). Studies have shown that CM polysaccharides and the butanol-soluble fraction of CI exhibit immunomodulatory effects and hold potential as therapeutic agents for infectious diseases (Xie et al., 2009; Sun et al., 2012; Liang et al., 2014; Li, 1993).

Inflammatory bowel disease (IBD) is an autoimmune disorder characterized by dysregulation of multiple immune-related pathways and cells, including the NF-κB signaling pathway, helper T (Th) cells, and regulatory T (Treg) cells (Chen et al., 2020; Committee FoCE, 1983; Chen Caiying et al., 2015; Committee FoCE, 2011). Numerous studies have demonstrated that inflammatory cytokine genes involved in IBD pathogenesis, such as IL-1β, IL-2, TNF-α, and IL-6, contain NF-κB binding sites and are transcriptionally regulated by NF-κB (Chen et al., 2020; Committee FoCE, 1983). Recent research has shown that CM polysaccharides can mitigate intestinal pathological changes in colitis rats, reduce levels of inflammatory cytokines (*e.g.*, TNF-α, IL-6, and IL-1β), and alleviate oxidative stress responses (*e.g.*, SOD and MPO). Furthermore, these effects are associated with decreased mRNA expression levels of TLR4, Janus kinase (JAK)2, and signal transducer and activator of transcription (STAT)3, as well as reduced protein levels of p65, TLR4, p-STAT3, and p-JAK2. Additionally, improvements were observed in the metabolic profiles of plasma and urine (Xie et al., 2009). All these findings indicate that the NF-κB/TLR4 and IL-6/JAK2/STAT3 signaling pathways play a role in the mechanism by which CM exerts its effects on IBD. Further studies have demonstrated that CM polysaccharides can reduce the production of pro-inflammatory cytokines and promote the production of anti-inflammatory cytokines by regulating the imbalance between Th1/Th2 and Th17/Treg (Sun et al., 2012). Additionally, improvements in gut microbiota have also been observed (Sun et al., 2012). However, while the effects of CM polysaccharides on IBD have been extensively studied, the depth of investigation is limited. Consequently, the specific mechanisms remain unclear, particularly regarding the rigorous validation of the signaling pathways involved.

Moreover, the immunomodulatory abilities of polysaccharides from different varieties of CM vary. Wang et al. (2022) investigated the immunomodulatory effects of polysaccharides from five cultivars: Qiju, Gongju, Boju, Hangbaiju, and Huaiju. The study found that all these polysaccharides could enhance the phagocytosis and proliferation of RAW264.7 cells without significant cytotoxicity, and upregulate the release of TNF-α, IFN-*γ*, and NO. Notably, polysaccharides from Boju and Hangbaiju exhibited superior immune-enhancing activities, making them more suitable for developing functional foods aimed at boosting immunity. These findings provide a reference for selecting appropriate varieties based on specific immune requirements, which are related to their relative molecular mass, glucuronic acid and arabinose content, and microstructure (Wang et al., 2022). Additionally, the potential role of these polysaccharides as vaccine adjuvants is currently under investigation (Han et al., 2021).

Additionally, the butanol soluble fraction of CI exhibited a significant inhibitory effect on dimethylbenzene-induced ear edema in mice and markedly enhanced the 2, 4-dinitro-fluorobenzene (DNFB)-induced delayed-type hypersensitivity (DTH) response (Cheng et al., 2005). Furthermore, CI was found to elevate the levels of sheep red blood cell (SRBC) antibodies, serum IgG, and IgM, while significantly enhancing the phagocytic function of monocytes in cyclophosphamide (CP)-induced immunosuppressed mice (Cheng et al., 2005). These findings indicate that CI possesses anti-inflammatory properties, as well as humoral and cellular immunomodulatory activities, including enhancement of mononuclear phagocytic function. The presence of flavonoids (53%) may contribute to these effects (Cheng et al., 2005).

Although the diseases examined in the aforementioned studies are not infectious in nature, the findings indicate that CM and CI possess certain immunomodulatory effects, which hold significant reference value for the subsequent management of infectious diseases. Further details are provided in Table 7.

**TABLE 7:** Detailed information of immunomodulatory effects of CM and CI.

Extracts/compounds	Dose	Vitro/vivo	Mode	Detail	Mechanism	Ref.
CM						
Polysaccharides (Aqueous extract)	50, 100 and 200 mg/kg	Vivo	TNBS/ethanol-induced colitis rats	In the colon tissue 1)TNF-α, IFN-γ, IL-6, and IL-1β production↓; NF-κB and IL-6 relative mRNA levels↓ 2)SOD↑, MPO↓, and MDA↓ activities 3)TLR4 (100 mg/kg), JAK2 (50 mg/kg), and STAT3 (50 and 100 mg/kg) mRNA expression levels↓; pp65, TLR4, p-STAT3, and p-JAK2 levels↓ 4)The metabolic profiles of plasma and urine were improved. (*p* < 0.05)	NF-κB/TLR4 and IL-6/JAK2/STAT3 and metabolic profiling signaling pathways	Tao et al. (2018)
Polysaccharides (95% ethanol extract)	50, 100 and 200 mg/kg	Vivo	TNBS/ethanol-induced colitis rats	1)The imbalance of Th1/Th2 and Th17/Treg↓: Th1: IFN-γ, IL-1β and TNF-α production↓; Th17: IL-6, IL-17 and IL-23 production↓; Th2: IL-13, IL-10 and IL-4 production↑ 2)Opportunistic pathogens *Escherichia, Enterococcus* and *Prevotella* abundance↓; protective bacteria such as *Butyricicoccus* and *Clostridium* (butyrate-producing bacteria), *Lactobacillus* and *Bifidobacterium* (probiotics), Lachnospiraceae and Rikenellaceae levels↑. (*p* < 0.05)	-	Tao et al. (2017)
Polysaccharides from five cultivars (ethanol extract)	12.5, 50 and 200 μg/mL	Vitro	RAW264.7 cells	1)The phagocytosis and proliferation of RAW264.7 cells↑ 2)TNF-α, IFN-*γ*, and NO production↑. (*p* < 0.001)	-	Wang et al. (2022)
CI						
A butanol soluble fraction (95% ethanol and butanol extract)	p.o.: 75, 150 and 300 mg/kg for 5 days i.p.: 50 mg/kg on day 3 and continued for 2 days	Vivo	1)Dimethylbenzene-induced mice 2)DNFB-induced mice 3)CP-induced mice	In dimethylbenzene-induced mice: Auricle tumidity↓. (150 mg/kg, p.o.) In DNFB-induced mice: DTH reactivity↑. (150 and 300 mg/kg, p.o.). In CP-induced mice: 1)Antibody generation by the splenic cells↑. 2)Serum IgG and IgM levels↑ in response to SRBC. 3)Macrophage phagocytic activity: the rate of carbon clearance↑ and phagocytic index↑. (150 and 300 mg/kg, p.o.) (*p* < 0.01)	-	Cheng et al. (2005)

Note: “↓”: Reduce or downregulate. “↑”: Increase or upregulate. “-”: not mentioned.

Oral administration (p.o.), intraperitoneal injection (i.p.).

### 3.5 Toxicology and safety profile

In the acute toxicity study, a single oral dose of 15 g/kg body weight (bw) CM extract was administered to rats, then the rats were observed for 14 days. No treatment-related death was observed, and the maximal tolerance dose estimated was greater than 15 g/kg bw in rats. For long-term toxicity studies, rats were given daily intragastric doses of 320, 640, and 1,280 mg/kg bw/day for 26 weeks, followed by a 4-week recovery period (Li et al., 2010). The results showed that there were no toxicological changes in body weight, food intake, water intake, blood biochemistry, organ weight and histopathological examination in each treatment group. Thus, CM extract is generally safe for rats at limited dose levels. Dosage of annotation in the ChP for 5–10 g (Commission CP, 2020).

Different doses of CI extract prepared in saline were given orally to groups of 10 mice. For 30 days subsequent to treatment, the animals were observed daily, and dead animals were subjected to *postmortem* examination for determination of the cause of death. No animals died during the acute toxicity test, nor were any adverse effects detected in animals treated with different doses of CI extract. This indicates that CI extract was nearly nontoxic in mice up to an oral dose of 2.0 g/kg body weight (Lee et al., 2009). No studies have investigated the long-term toxicity of CI. Dosage of annotation in the ChP for 9–15 g (Commission CP, 2020). In addition to oral administration, it can also be used for external use, decoction for washing or paste for external application.

## 4 Conclusion and future perspectives

CM and CI, two major traditional Chinese herbs recognized in the theory of food and medicine homology, have a long history of medicinal use and demonstrated significant clinical efficacy. By systematically reviewing literature from the perspectives of ethnopharmacology, phytochemistry, and pharmacology, this study aims to provide an updated understanding of the anti-infective effects of CM and CI, thereby facilitating a more comprehensive grasp of their potential clinical applications and current research status in the context of infectious diseases.

A 2020 review reported that over 176 compounds, including 60 flavonoids, 28 phenylpropanoids, 68 triterpenoids, three steroids, and 17 others, were isolated and identified in CM. Notably, the “other” category included a small number of terpenoids (only 20 compounds) (Yuan et al., 2020). Subsequently, Peng et al. (Peng et al., 2020) detected a significant number of terpenoids, primarily monoterpenes, from CM, thereby supplementing the earlier review. Overall, a total of 109 terpenoids, including monoterpenoids, sesquiterpenoids, and unidentified diterpenes, have been identified in CM. Additionally, it has been reported that 191 natural compounds were isolated and identified from CI, comprising 42 flavonoids, 96 terpenoids, 21 phenylpropanoids and phenolic acids, 12 spiro ketones, and 20 other compounds (Shao et al., 2020). In this study, we summarize the main compounds reported in studies on the anti-infection-related antimicrobial, anti-inflammatory, antioxidant, and immunomodulatory effects of CM or CI, including 53 alkenes and terpenoids, 11 flavonoids, 7 phenylpropanoids, and other compounds. These compounds play a crucial role in inhibiting microbial growth, reducing inflammation, combating oxidative stress, and modulating immune responses.

Although CM has been reported to contain a higher number of compounds compared to CI, CI has shown superior antimicrobial effects in certain studies (Youssef et al., 2020). This may be attributed to the high concentration of key compounds in CI. For instance, camphor (No.8), a bicyclic monoterpene, exhibits a wide range of biological activities, including insecticidal, analgesic, antimicrobial, antiviral, anticoccidial, antinociceptive, anticancer, and antitussive properties (Sokolova et al., 2015; Zielińska-Błajet and Feder-Kubis, 2020). Studies have shown that symmetrical compounds containing camphor fragments, two imino groups, and/or a charged quaternary nitrogen atom exhibit potent antiviral activity (Sokolova et al., 2014; Sokolova et al., 2013). Additionally, research evaluating the antiviral activity of camphor-based imine derivatives against the H1N1 influenza virus pdm09 and their inhibitory effects on H5N1 pseudovirus infection revealed that the critical structural units responsible for antiviral activity include the natural camphor skeleton, the presence of an imino group, and an alcohol moiety (Sokolova et al., 2015).

A study demonstrated that CI and CM exhibited limited antifungal efficacy, whereas another study indicated that CM displayed superior antifungal properties (Youssef et al., 2020; Zhang et al., 2020). The discrepancy may be attributed to the different plant parts used for CM extraction; the former study utilized flowers, while the latter employed roots. Specifically, the latter study analyzed 12 cultivars of CM roots, revealing that the primary compounds in root extracts differ from those in flower extracts, which likely explains the varying antifungal effects observed. Additionally, differences in extraction methods and fungal strains examined between the studies may have contributed to these discrepancies. Regarding antiviral activity, CI showed a stronger inhibitory effect on specific viruses such as VSV, HAV, and HSV-1 (Youssef et al., 2020; Gu et al., 2013). However, some studies reported antimicrobial activity using only IC50 values without MIC or *vice versa*, potentially introducing bias into their results.

Furthermore, both CM and CI extracts exhibit anti-inflammatory properties. CM can regulate inflammation by modulating the AMPK-SIRT1 and TLR4 signaling pathways (Lee et al., 2021; Zeng et al., 2020). CI modulates inflammatory pathways, including the TLR4/MyD88-dependent NF-κB signaling pathway, and MAPKs and NF-κB-dependent pathway, as well as ASC phosphorylation independent of JNK phosphorylation (Yu et al., 2019; Wu et al., 2014; Cheon et al., 2009). Both CM and CI extracts reduce oxidative stress markers such as MDA and increase antioxidant enzyme activities, including SOD, CAT, and GPX (Lii et al., 2010; Zhan et al., 2022; Hao et al., 2021; Tian et al., 2019; Zhang et al., 2019; Kim et al., 2017). The anti-oxidative mechanism of CM involves the arginine and purine metabolic pathways, the PI3K/Akt-mediated Nrf2/HO-1 signaling pathway, and others (Lii et al., 2010; Zhan et al., 2022; Hao et al., 2021; Tian et al., 2019). CM polysaccharides have demonstrated immunomodulatory effects in multiple studies, potentially through the NF-κB/TLR4 and IL-6/JAK2/STAT3 pathways, as well as by balancing Th1/Th2 and Th17/Treg cells (Tao et al., 2018; Tao et al., 2017; Wang et al., 2022). CI can regulate the immune response, which may be attributed to its flavonoid content (Cheng et al., 2005).

In TCM, infectious diseases are often conceptualized as disruptions to the body’s balance caused by pathogenic factors such as “heat”, “dampness”, and “wind” (Wu et al., 2023). To eliminate these influences, botanical drugs with properties that “clear heat” and “detoxify” are utilized (Xu et al., 2019; Fang et al., 2009). The objective is to restore balance and harmony within the body, thereby alleviating both the symptoms and underlying causes of the disease. Specifically, CM and CI are used for their heat-clearing and detoxifying attributes, which effectively eliminate these pathogenic factors (Commission CP, 2020). CM and CI are commonly employed in TCM to alleviate symptoms of infections, including fever, inflammation, swelling, and abscesses, which align with the TCM concepts of “heat” and “toxins”. In contemporary pharmacological research, CM and CI extracts have been shown to effectively alleviate infection severity and prevent associated complications by modulating inflammatory responses, enhancing immune function, and mitigating oxidative stress. However, our review reveals that research on CI’s antimicrobial and anti-inflammatory properties is likely more extensive compared to that on CM, indicating that CI may hold greater potential for treating infectious diseases. Additionally, the efficacy of CM and CI in TCM formulations warrants further attention. Sangju cough mixture, which includes CM, has demonstrated therapeutic efficacy against colds. It can also alleviate symptoms of *mycoplasma* pneumoniae (MP), expedite the negative conversion time of MP-IgM antibodies, and facilitate patient recovery (Yang et al., 2024; Ji et al., 2019). In addition, Xiasangju is a well-known TCM formula for treating fever and influenza, and its composition includes CI. Increasing evidence suggests its various pharmacological effects on bacterial infections, immune system disorders, and other conditions (Wu et al., 2022). Overall, CM and CI serve as prime examples of how traditional botanical knowledge can be integrated into contemporary therapeutic practices, providing valuable treatments for infectious diseases through their heat-clearing and detoxifying properties.

Moreover, we have not identified any clinical study reports on the use of CM and CI. In TCM, treatments typically involve TCM formulas rather than single botanical drugs. Therefore, conducting clinical studies using only single botanical drug may present challenges. Future research could focus on the application of traditional Chinese medicinal formulas that include CM and CI for treating infectious diseases (Yang et al., 2024; Ji et al., 2019; Wu et al., 2022).

However, comprehensive comparative studies between CM and CI are imperative. Rigorous investigations are required to elucidate the precise molecular mechanisms underlying their antimicrobial, anti-inflammatory, immunomodulatory, and antioxidant effects, including the interactions of specific compounds with cellular targets and signaling pathways. Furthermore, potential synergistic effects among different compounds within CM and CI warrant exploration. Additionally, both CM and CI extracts have demonstrated promising effects in preliminary studies, and further investigations, such as pharmacokinetic studies, could enhance our understanding of their bioavailability. Finally, establishing robust quality control measures and standardization protocols for CM and CI extracts will ensure consistent efficacy and safety in therapeutic applications, ultimately aiding in the fight against infectious diseases.
